# Caregiver burden is increased in Parkinson’s disease with mild cognitive impairment (PD-MCI)

**DOI:** 10.1186/s40035-017-0085-5

**Published:** 2017-06-19

**Authors:** Ann J. Jones, Roeline G. Kuijer, Leslie Livingston, Daniel Myall, Kyla Horne, Michael MacAskill, Toni Pitcher, Paul T. Barrett, Tim J. Anderson, John C. Dalrymple-Alford

**Affiliations:** 1 New Zealand Brain Research Institute, 66 Stewart Street, Christchurch, 8011 New Zealand; 2 Department of Psychology, University of Canterbury, Christchurch, New Zealand; 3 Department of Medicine, University of Otago, Christchurch, New Zealand; 4 Cognadev, UK Ltd., Sandton, South Africa; 5 Department of Neurology, Christchurch Hospital, Christchurch, New Zealand; 6 Brain Research New Zealand Centre of Research Excellence, Christchurch, New Zealand

**Keywords:** Parkinson’s disease, Mild cognitive impairment, Zarit caregiver burden interview, Coping, Depression, Anxiety, Positive aspects of caregiving

## Abstract

**Background:**

There is limited evidence on caregiver outcomes associated with mild cognitive impairment in patients with Parkinson’s disease (PD-MCI) and the coping strategies used by these caregivers.

**Methods:**

To investigate this relationship, we examined levels of burden, depression, anxiety, coping strategies and positive aspects of caregiving in the informal caregivers of 96 PD patients. The PD patients were classified using MDS-Task Force Level II criteria as showing either normal cognition (PD-N; *n* = 51), PD-MCI (*n* = 30) or with dementia (PDD; *n* = 15).

**Results:**

Mean Zarit Burden Interview (ZBI) score increased significantly between carers of PD-N (M = 13.39, SD = 12.22) compared to those of PD-MCI patients (M = 22.00, SD = 10.8), and between carers of PD-MCI and PDD patients (M = 29.33, SD = 9.59). Moreover, the proportion of carers showing clinically significant levels of burden (ZBI score ≥ 21) also increased as the patients’ cognitive status declined (18% for PD-N; 60% for PD-MCI; and 80% for PDD) and was mirrored by an increasing amount of time spent providing care by the caregivers. Caregiver ZBI score was independent of patient neuropsychiatric symptoms, motor function, disease duration and time that caregivers spent caregiving. Caregiver use of different coping strategies increased with worsening cognition. However, we found only equivocal evidence that the use of problem-focused, emotion-focused and dysfunctional coping mediated the association between patient cognitive status and caregiver burden, because the inverse models that used caregiver burden as the mediator were also significant.

**Conclusions:**

The study highlights the impact of Parkinson’s disease on those providing care when the patient’s cognition is poor, including those with MCI. Caregiver well-being has important implications for caregiver support, nursing home placement and disease course.

**Electronic supplementary material:**

The online version of this article (doi:10.1186/s40035-017-0085-5) contains supplementary material, which is available to authorized users.

## Background

Cognitive problems are an integral part of Parkinson’s disease because dementia (PDD) eventuates in 75%–90% of patients and is associated with substantial health and economic burden [1]. Patients who present with mild cognitive impairment (PD-MCI) are at high risk of progression to PDD and are a growing research focus to assess the impact of increasing cognitive impairment and to facilitate early intervention [2–5]. The impact of cognitive status on the informal caregiver is a relatively neglected dimension of PD-MCI and there is limited research on the specific effects of cognitive decline on the well-being of caregivers of these PD patients. Understanding these relationships is important because improving caregiver support and well-being may have a bearing on disease management and delay formal care and nursing home placement.

It is well-established that there is higher caregiver burden and reduced quality of life in caregivers once dementia onset has commenced in PD [6, 7]. Recent assessments of well-being in PD-MCI caregivers has revealed mixed results, in that reduced quality of life measures were found in these caregivers [8] but explicit measures of burden using the Zarit Burden Interview (ZBI), did not show elevated levels of burden for PD-MCI caregivers as compared to PD-N caregivers [9, 10]. Such results contrast with reports of increased caregiver burden in carers of people with MCI who are at risk of Alzheimer’s dementia [11], although additional factors may contribute to PD caregiver difficulties [12]. The failure to find increased Zarit burden scores in carers of PD-MCI patients is unexpected, but may reflect the over-riding impact of motor disabilities and other nonmotor symptoms in PD even prior to dementia.

The Zarit Burden Interview provides a well-validated global measure of the physical, emotional and socioeconomic impact of caring for neurologically compromised individuals [13]. While motor and neuropsychiatric symptoms present one explanation of the response of the caregiver to the patient with PD, an alternative reason for the failure to identify increased burden with PD-MCI patients may be that caregiving strategies develop within the context of ostensibly a movement disorder that has a dominant influence on the interaction between patient and caregiver. For example, the response by caregivers to the presence of MCI in the context of an already established disease such as PD may evoke compensatory positive attitudes in carers or may influence their coping strategies before problems associated with dementia become overwhelming. In the wider literature in the context of dementia, the use of three coping strategies (problem-focused, emotion-focused and dysfunctional coping) has been found useful to explain variability in caregiver outcomes in that dementia caregivers who use more emotion-focused coping and less dysfunctional coping express lower anxiety and depression [14]. Dysfunctional strategies include substance use, distraction, and disengagement, while emotion-focused coping concerns attempts to reduce distress by regulating emotions which may prove adaptive in the context of PD caregiving. Problem-focused coping relates to managing distress through confronting and altering the situation, but findings for this strategy are less clear [14]. Identifying these strategies has the benefit of providing a focus for interventions to improve caregiver well-being [15, 16]. We therefore examined caregiver coping strategies and positive aspects of caregiving as potential mediators of the relationship between patients’ cognitive status and caregiver burden.

In the current study we employed a cross-sectional design to examine burden expressed by caregivers of patients who met the current Movement Disorders Society – Task Force (MDS-TF) Level II criteria for PD-MCI and PDD [17, 18] and by caregivers of patients with normal levels of cognition (PD-N). Previous studies that assessed caregivers of PD-MCI patients employed Level I criteria, in which the range of tests employed are restricted either to global measures of cognition or a relatively restricted range of cognitive measures [8–10]. We have found that specific Level II criteria, in which two impairments are required within a single domain, capture PD-MCI patients who are at greatest risk of decline to PDD in the subsequent 4 years [4] and who may thus pose a greater challenge to caregiver well-being. The MDS-TF level II criteria also enabled an assessment of caregiver outcomes associated with PD-MCI subtypes [10] including attentional deficits, which have been shown to be associated with lower Quality of Life in PD carers [8].

We hypothesized that caregivers of patients meeting Level II PD-MCI criteria would express a level of burden (ZBI) that was intermediate to that experienced by PD-N and PDD caregivers. It was also anticipated that attentional deficits in PD patients would be associated with increased caregiver burden [10]. Further, it was hypothesized that, similar to Alzheimer’s caregivers, [19] use of emotion-focused-coping strategies would reduce this level of distress (ZBI) but that use of problem-focused coping and dysfunctional coping would not.

## Methods

A convenience sample of 96 PD patients (UK Parkinson’s Society criteria), part of a longitudinal study, were recruited through the New Zealand Brain Research Institute. Caregivers were identified as any person who was directly involved in the patient’s care and provided some form of support with respect to everyday activities. These caregivers either lived with the patients (*n* = 82), were spouses, partners or children and spent a minimum of 4 hours per week caring for the patient. At the time when caregivers were interviewed for this study, 15 patients met criteria for PDD, while the remaining PD patients were assessed as showing either PD-MCI (*n* = 30) or cognitive abilities within a normal range of scores (PD-N; *n* = 51). The PD patients met MDS-TF level II criteria by completing neuropsychological assessments over 2 sessions using 23 measures across the recommended five cognitive domains (attention, working memory and processing speed; executive function; visuoperceptual/visuospatial; learning and memory; language) within 6 months of the caregiver burden and coping assessments. A diagnosis of PD-MCI was confirmed when any two (or more) impaired neuropsychological test scores were present within any single domain but everyday independent function as reported by the caregiver [20] was generally preserved and indicated cognitive independence. A score 1.5SD or more below normative data was considered an impaired score [4]. Evidence of PDD was determined when the caregiver reported inability by the patient on everyday tasks in the context of impaired cognition in two or more cognitive domains [20], which was supplemented by assessment of the patient’s performance during interview and caregiver responses on the neuropsychiatric inventory (NPI) [21]. Motor function was assessed with the Unified Parkinson’s disease Rating Scale part III (UPDRS-III) [22]. All patients were in an ‘on’ state during neuropsychological assessment. Table 1 provides individual neuropsychological test and domain scores for the three patient groups. Caregiver demographical information and clinical characteristics of the caregivers and patients are provided in Tables 2 and 3.

**Table 1:** Neuropsychological assessments used for level II criteria (*n* = 96)

Patient groups	PD-N(*n* = 51)	PD-MCI(*n* = 30)	PD-D(*n* = 15)	Analysis (*F*_*2,93*_)
MoCA^a^	26.64 ± 2.16	24.53 ± 3.08	21.33 ± 2.32	27.12, *p* < .0001
Attention, Working Memory and Processing Speed				
DigitsF/B	.54 ± 0.93	.14 ± 0.69	−.37 ± 0.64	7.69, *p* < .01
Digit Ordering	−.60 ± 1.06	−1.33 ± 1.10	−2.16 ± 0.89	13.52, *p* < .001
TEA (Map Search)	−.46 ± 0.84	−1.40 ± 0.85	−1.91 ± 0.72	23.6, *p* < .001
Stroop colour	.09 ± 0.80	−.60 ± 1.14	−1.13 ± 1.07	15.03, *p* < .001
Stroop word	.15 ± 0.73	−.34 ± 1.06	−.68 ± 1.07	6.17, *p* < .01
Trails A	.32 ± 0.69	−.41 ± 0.99	−1.7 ± 1.22	8.83, *p* < .001
Domain Score	.01 ± 0.50	−.65 ± 0.51	−1.40 ± 0.52	47.78, *p* < .001
Domain Pass/Fail	51/0	13/17	2/13	
Executive Function				
Letter Fluency	.61 ± 1.45	.28 ± 1.24	−.42 ± 1.40	3.32, *p* < .05
Action Fluency	−.78 ± 1.08	−1.45 ± 1.09	−1.76 ± 1.03	6.56, *p* < .01
Category Fluency	.68 ± 1.14	.07 ± 1.09	−.95 ± 1.17	12.55, *p* < .001
Category Switching	.27 ± 1.12	−.52 ± 1.27	−1.95 ± .89	22.80, *p* < .001
Trails B	.28 ± 0.83	−.30 ± 1.13	−2.31 ± 0.84	44.03, *p* < .0001
Stroop Interference	.31 ± 1.04	−.45 ± 0.78	−1.66 ± 0.70	35.01, *p* < .0001
Domain Score	.23 ± 0.74	−.40 ± 0.72	−1.05 ± 0.55	36.05, *p* < .0001
Domain Pass/Fail	51/0	15/15	2/13	
Learning & Memory				
CVLT Free recall	.59 ± 1.13	−.29 ± 0.96	−1.44 ± 0.90	23.26, *p* < .001
CVLT Short delay	.31 ± 1.32	−.33 ± 1.28	−1.43 ± 0.53	12.17, *p* < .001
CVLT Long delay	.38 ± 0.90	−.23 ± 0.98	−.76 ± 0.53	11.38, *p* < .001
Rey Immediate	.59 ± 1.71	−.56 ± 1.08	−1.34 ± 1.03	12.84, *p* < .001
Rey Delayed	.58 ± 1.77	−.85 ± 1.15	−1.82 ± 1.08	18.13, *p* < .001
Domain Score	.50 ± 1.06	−.45 ± 0.80	−1.41 ± 0.46	28.50, *p* < .0001
Domain Pass/Fail Visuospatial	51/0	24/6	4/11	
JOL	−.15 ± 0.81	−.99 ± 0.81	−.94 ± 0.84	10.55, *p* < .001
Fragmented letters	.62 ± 0.58	.10 ± 9.99	.20 ± .08	7.9, *p* < .05
Rey Copy	.02 ± 1.04	−.85 ± 1.27	−1.61 ± 1.11	14.11, *p* < .01
Domain Score	.44 ± 0.55	−.31 ± 0.63	−.76 ± 0.57	31.94, *p* < .0001
Domain Pass/Fail	51/0	26/4	11/4	
Language				
Boston Naming	.21 ± 0.86	.05 ± 1.07	−.11 ± 1.21	*F*_2,64_ = .87, *p* = .42
ADAS-Cog	.01 ± 0.65	−.18 ± 0.78	−1.16 ± 0.59	14.89, *p* < .001
DRS-2	.01 ± 0.57	−.18 ± 0.66	−.62 ± 0.88	5.45, *p* < .01
Domain Score	.06 ± 0.47	−.10 ± 0.46	−.67 ± 0.57	13.64, *p* < .001
Domain Pass/Fail	51/0	29/1	14/1	
Global neuropsychological *z* score	.29 ± 0.57	−.45 ± 0.41	−1.29 ± 0.41	62.23, *p* < .001

Values reported as mean ± standard deviation

^a^MoCA Montreal Cognitive Assessment, with one missed test; Age and education z scores for all tests except MoCA; global performance was expressed by an aggregate *z* score by first averaging standardized scores within four cognitive domains and then taking the mean of these four values; the language domain scores were not included in this *z* score due to the distributions of the normative data

**Table 2:** Demographic and clinical characteristics of the Parkinson’s disease patient groups *n* = 96

Patient groups	PD-N (*n* = 51)	PD-MCI (*n* = 30)	PDD (*n* = 15)	Analysis	Significant post-hoc differences (N-K or T2)
Age	68.23 ± 7.60	69.5 ± 6.86	72.73 ± 4.85	F_2,93_ = 2.39, *p* = .09	
Female: Male	16/35	9/21	3/12	*X* ^2^ = .74, *p* = .69	
Education (yrs)	13.14 ± 3.03	12.56 ± 1.99	12.26 ± 2.49	F_2,93_ = .82, *p* = .44	
GDS	1.45 ± 2.88	0.73 ± 1.79	2.13 ± 3.14	F_2,93_ = 1.29, *p* = .28	
H&Y	2.10 ± 0.52	2.30 ± 0.75	2.33 ± 0.59	F_2, 90_ = 1.48, *p* = .23	
NPI	3.14 ± 5.21	4.37 ± 4.62	8.13 ± 9.26	F_2, 89_ = 4.83, *p* < .05	PD-N v PDD, *p* < .05
ADL-IS	.51 ± 0.51	.78 ± 0.47	2.04 ± 0.54	F_2,91_ = 55.35, *p* < .0001	PD-N v PDD, *p* < .001 PD-MCI v PDD, *p* < .001
Disease Duration (yrs)	7.54 ± 4.00	9.10 ± 4.44	11.63 ± 6.92	F_2, 93_ = 4.59, *p* < .05	
UPDRS-III	25.64 ± 10.80	29.2 ± 13.07	34.83 ± 10.42	F_2,92_ = 3.84, *p* < .05	PD-N v PDD, *p* < .05

Values reported as mean ± standard deviation: GDS-Geriatric Depression Scale; H&Y- Hoehn & Yahr; NPI - Neuropsychiatric Inventory; ADL-IS Activities of Daily Living - International Scale (max = 4.0); UPDRS-III Unified Parkinson’s Disease Rating Scale Part III

**Table 3:** Demographic and clinical characteristics of the caregivers (*n* = 96)

Caregivers of patient groups	PD-N (*n* = 51)	PD-MCI (*n* = 30)	PDD (*n* = 15)	Analysis	Significant post-hoc differences (N-K or T2)
Age	65.53 ± 9.68	62.33 ± 13.61	66.87 ± 11.34	F_2,93 =_ 1.07*, p* = .35	
Female/Male	33/18	21/9	12/3	*X* ^2^ = 0.74, *p* = .36	
Years Education	12.41 ± 2.36	12.33 ± 2.45	11.60 ± 1.88	F_2,93 =_ 0.73, *p* = .49	
Spouse/Other^a^	47/4	24/6	12/3	*X* ^2^ = 3.02, *p* = .22	
Lives separately	3	6	5	*X* ^2^ = 8.04*, p* = .98	
Hours/week caregiving	5.40 ± 14.30	16.47 ± 27.06	25.01 ± 27.23	F_2,93_ = 10.17, *p* < .001	PD-MCI v PDD, *p* < .05 PD-N v PDD, *p* < .001
Zarit Burden Interview	13.39 ± 12.22	22.00 ± 10.86	29.33 ± 9.59	F_2,93_ = 13.89, *p <* .001	PD-N v PD-MCI, *p* < .01 PD-N v PDD, *p* < .001 PD-MCI v PDD, *p* < .05
Coping Strategies					
Problem-Focused	1.88 ± .67	2.20 ± .62	2.35 ± .65	F_2,93_ = 4.09, *p* < .05	PD-N v PDD, *p* < .05
Emotion-Focused	1.93 ± .55	2.24 ± .64	2.27 ± .47	F_2,93_ = 3.61, *p* < .05	
Dysfunctional	1.28 ± .40	1.43 ± .38	1.55 ± .37	F_2,93_ = 3.32, *p* < .05	PD-N v PDD, *p* < .05
GDS	1.21 ± 1.94	0.73 ± 1.41	0.40 ± .83	F_2,93_ = 1.59, *p* = .21	
GAI	2.47 ± 3.85	2.47 ± 3.81	0.80 ± 1.61	F_2,93_ = 1.52, *p* = .22	
Positive Aspects of Caregiving	27.12 ± 8.85	27.316 ± 9.20	24.87 ± 9.36	F_2,93_ = 0.40, *p* = .67	

Values reported as mean ± standard deviation

^a^Daughter, son, daughter-in-law, brother or friend

### Measures

#### The Zarit burden interview (ZBI)

This 22-item scale identifies the impact of the patient’s disability in terms of caregiver health, finances, emotion, social life and interpersonal relations [13]. For each item, the caregiver rates how often they have felt the suggested feeling or perception, from never (score 0) to nearly always (score 4), generating a score ranging from 0 to 88. In dementia caregivers, the measure has good internal consistency with a Cronbach’s alpha of .92. A score ≥ 21 signifies mild to moderate burden [13].

#### The brief coping orientations for problems experienced (COPE)

This 28-item version of the 60-item inventory assesses coping strategies [23]. Caregivers were asked to consider their current PD-caregiving situation. Responses were scored on a 4-point Likert scale ranging from ‘I usually don’t do this at all’ through to ‘I usually do this a lot’. Principal component factor analysis identified three dimension scores involving problem-focused, emotion-focused and dysfunctional coping (Additional file 1: Table A and Table B).

#### Positive aspects of caregiving (PAC)

This 9-item instrument provided a positive dimension score of the caregiving experience [24].

#### Depression/anxiety

The 15-item Geriatric Depression Scale (GDS) [25] was used to screen for depressive symptoms in caregivers and patients while anxiety symptoms in caregivers were assessed with the Geriatric Anxiety Inventory (GAI) [26].

#### Hours per week spent caregiving

Caregivers were asked to estimate the average amount of time they spent each day providing care to the PD patient, which was multiplied by 7 to give a total of hours per week. Caregivers were asked to consider the provision of care relating to PD symptoms.

#### Neuropsychiatric inventory

The Neuropsychiatric Inventory (NPI) [21] assesses 10 behavioural disturbances: delusions, hallucinations, dysphoria, anxiety, agitation/aggression, euphoria, disinhibition, irritability/lability, apathy, and aberrant motor activity. Information for the NPI is obtained from the caregiver about the patient’s behaviour. Only those domains with positive responses to screening questions are used for scoring. The frequency of the symptoms is scored on a 4-point scale, severity on a 3-point scale, and distress caused by the patient’s symptoms on a 5-point scale.

#### Activities of daily living – International scale

The Activities of Daily living – International scale (ADL-IS) [20] consists of 40 questions such as “Does [the patient] have difficulty putting household items in the right places?” to which the informant is asked to respond using a Likert scale of 0 = ‘never has difficulty’ to 3 = ‘always has difficulty’. A response of 4 = ‘activity no longer performed (ie. has given up initiating the activity)’; 8 = ‘activity was never performed’ and 9 = ‘unknown’. A score between 0 and 4 is generated based on the number of items that the patient was known to perform prior to their illness.

### Statistical analysis

All variables in the study were screened for outliers and deviations from normality. Single outliers were detected for disease duration, Hoehn & Yahr score, caregiver age and the ZBI. Since none of the results changed when outliers were excluded from analysis, it was decided to retain these data points. The distribution of the GDS (patient and caregiver), the NPI, hours of caregiving and the GAI violated assumptions of normality. A log transformation was used to ensure that skewness and kurtosis of these variables were within the acceptable range (means and standard deviations prior to transformation are presented in the tables to assist interpretability). Comparison of demographic and clinical variables across the three cognitive status groups, for both patients and caregivers, was assessed using Analysis of Variance (ANOVA) and Chi square. Newman-Keuls (N-K) tests for equal group variances and Tamhane’s T2 (T2) tests for unequal group variances were used for post-hoc comparisons. Additional analyses of covariance examined whether any effects of cognitive status on caregiver burden remained after controlling for patient clinical variables and time spent caregiving. Pearson’s correlations between PD patient cognitive status (defined as 1 = PD-N, 2 = PD-MCI, 3 = PD-D) [27], caregiver burden and the three coping strategies were assessed to see whether criteria for path analysis (mediation) were met. The PROCESS macro developed by Hayes [28] was used to test for mediation using linear regression. In this procedure the mediated effect (indirect effect) is calculated via bootstrapping using a 95% confidence interval (CI) [29]. A mediator is significant when the 95% CI does not include zero. Separate analyses for each of the three coping strategies as intended mediators were conducted. Reverse causation, with caregiver burden as the mediator, was also assessed. All analyses employed an alpha level of *p* < 0.05 and were two-tailed.

## Results

Patients in the 3 cognitive status groups did not differ significantly in mean age, years of education, depression symptoms or disease stage (Table 2). There were, however, significant differences between the three groups in neuropsychiatric inventory (NPI) symptoms, abilities at everyday tasks (ADL-IS score), motor features (UPDRS III score) and disease duration. Scores for these patient measures increased linearly from PD-N to PD-MCI through to PDD, with significant differences between the PD-N versus the PDD groups on all measures apart from disease duration, and between PD-MCI and PDD on the ADL-IS.

The caregivers were aged between 23 and 83 years old, and predominantly a slightly younger spouse (85.4%) and female (70.8%). Caregivers of the PD patients in the three cognitive status groups did not differ in mean age, years of education, positive aspects of caregiving, depression or anxiety symptoms (Table 3). Caregiver burden (ZBI), however, increased significantly across the 3 cognitive status groups (F_2, 93_ = 13.89, *p* < .00001) with post-hoc tests (N-K) showing significant differences between all 3 groups. Moderate to large effect sizes were found for the differences between PD-N versus PD-MCI (Cohen’s *d* = .73; [CI = .27, 1.20]) and between PD-MCI versus PDD (Cohen’s *d* = .70; [CI = .064, 1.33]). A large effect size was found for the difference between PD-N versus PDD (Cohen’s *d* = 1.36; [CI = .74, 1.98]). The proportion of carers showing mild to moderate burden (ZBI ≥ 21) [13] was 18% for PD-N, 60% for PD-MCI and 80% for PDD; these proportions were significantly different to chance (χ^2^ = 32.45, *p* < .0001). There were no significant correlations between caregiver ZBI or coping strategies and any of the five individual cognitive domain scores used to assess the PD-MCI group. Mean caregiver ZBI scores did not differ significantly when PD-MCI patients were subdivided according to possible cognitive subtypes: PD-MCI with attention deficit only, *n* = 10, M = 20.9 (SD = 12.88); executive only, *n* = 8, M = 24.15 (SD = 6.22); memory only, *n* = 4, M = 25.25 (SD = 20.13); visuoperception only, *n* = 1; and any multidomain impairments, *n* = 7, M = 19 (SD = 8.18). Lack of clear effects of multidomain were also evident when the multidomain classification focused on either attention plus any other domain, *n* = 7, M = 19 (SD = 8.18) or executive plus any other domain, *n* = 7, M = 19 (SD = 8.18), although there was some suggestion of less burden than the multidomain impairment derived from memory plus any other domain, *n* = 2, M = 8.5 (SD = .71) and visuoperceptual plus any other domain, *n* = 3, M = 15.33 (SD = 5.5).

The number of hours that caregivers spent per week caring for PD patients also increased significantly for caregivers across the 3 cognitive status groups (Table 3). Post-hoc tests (T2) confirmed significant differences in time spent caregiving between PD-MCI versus PDD: medium to large effect size, Cohen’s *d* = .74 [CI = .09, 1.37]) and PD-N versus PDD: large effect size, Cohen’s *d* = 1.49 [CI = .86, 2.11), but the difference between PD-N versus PD-MCI did not reach significance: Cohen’s *d* = .43 [CI = −.04, .88].

Additional covariance analyses showed that the significant effect of patient cognitive status on the ZBI scores remained after controlling for disease duration (F(2,92) = 9.16, *p* < .001), patient neuropsychiatric symptoms [NPI] (F(2, 88) = 16.04, *p* < .001), ability to perform everyday tasks [ADL-IS] (*F*(2, 88) = 6.70 *p* < .05), motor difficulties (UPDRS-III) (F(2, 91) = 12.74, *p* < .001) and even time spent caregiving (F(2, 92) = 9.09, *p* < .001). There was no difference in ZBI scores between the caregivers of PD-MCI patients who converted to PDD within 2 years of the caregiver interview compared to those who did not convert *t*(5) = 1.8, *p* = .13. There was also no difference in ZBI scores between female (M = 19.26, SE = 1.58) and male (M = 16.17, SE =2.15) caregivers (*t* = 1.15, *p* = 2.00) and no significant interaction between the effects of patient cognitive status and gender of caregiver (*F*(2, 92) = .60, *p* = .43).

One aim of this study was to examine whether coping strategies may provide a mechanism to explain variability in caregiver outcomes. There were significant differences across the groups in the use of problem-focused, emotion-focused and dysfunctional coping (Table 3). Post-hoc tests (N-K) identified significant differences between PD-N and PDD caregivers only for problem-focused coping (Cohen’s d = .71[CI = .12, 1.29]) and dysfunctional coping (Cohen’s d = .69 [CI = .10, 1.27]). There were no differences between the three caregiver groups in terms of positive aspects of caregiving.

**Fig. 1:**
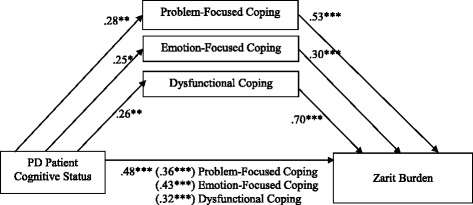
Standardized regression coefficients for the relationship between PD patient cognitive status and caregiver burden as mediated by use of problem-focused, emotion-focused and dysfunctional coping strategies (*n* = 96). The standardized regression coefficients between cognitive status and burden controlling for coping strategies are in parentheses *p < .05, **p < .01, ***p < .001

Correlational analyses identified that all 3 coping strategies correlated with both cognitive status and with caregiver ZBI score and thus met criteria for path analysis (Table 4). Support for (partial) mediation requires that the relationship between cognitive status and caregiver burden decreases significantly once the mediator is added to the regression model. A mediator is significant when the 95% CI of the mediated effect does not include zero. The bootstrapping procedure provided support for mediation when problem-focused coping was used as the intended mediator, (*b* = 2.02, *SE* = 0.97, [CI = 0.58, 4.47]). The same was true for the analyses with emotion-focused coping as the intended mediator (bootstrapping procedure: *b* = 0.82, *SE* = .59, [CI = .06, 2.47]) and dysfunctional coping (*b* = 2.70 SE = 1.04 [CI = .85–4.94]). Thus, problem-focused coping, emotion-focused coping and dysfunctional coping all partially mediated the relationship between patients’ cognitive status and caregiver burden (Fig. 1). However, given the cross-sectional nature of our design, it was also necessary to examine the reverse models in which caregiver burden (ZBI) may instead mediate the relationship between cognitive status and problem-focused coping, emotion-focused coping and dysfunctional coping. This reverse analysis was significant for all three coping measures: problem-focused: *b* = .22 *SE* = .06 [CI = .12, .37], emotion-focused: *b* = .09, *SE* = .06 [CI = .01, .24], and dysfunctional: *b* = .19, *SE* = .03 [CI = .13, .27]. Given these bidirectional mediating relationships, we cannot be certain that coping strategies mediated the association between patient’s cognitive status and caregiver ZBI score.

**Table 4:** Pearson’s correlations between caregiver burden, cognitive status, and coping strategies (*n* = 96)

	ZBI	CogSt	P-F Coping	E-F Coping
CogSt	0.48***			
P-F Coping	0.53***	0.28**		
E-F Coping	0.30***	0.25*	0.41***	
Dysf Coping	0.70***	0.26**	0.41***	0.26**

*ZBI* Zarit burden interview, *CogSt* Cognitive status, *P-F* Problem-focused, *E-F* Emotion-focused, Dysf = dysfunctional. *p < .05, **p < .01, ***p < .001

## Discussion

Assessing the impact of PD-MCI on patients and caregivers is seen as a ‘crucial unmet need’ [18] but has been scarcely studied. PD patients in the present study were classified as PD-N, PD-MCI or PDD according to level II criteria. Unlike previous work using Level I criteria, we identified increased burden among carers of PD-MCI patients compared with those of PD-N. Further, PDD caregivers had significantly higher ZBI scores compared to both PD-N and PD-MCI caregivers. The use of all three coping strategies increased as the patient’s cognitive status worsened. There were no differences in terms of positive caregiving aspects, depression or anxiety across the carers of the different PD patient groups. The current study’s ZBI findings on burden in caregivers of PD-MCI patients are in contrast to previous research by Leroi et al. [9] and Szeto et al. [10].

Leroi and colleagues identified moderate burden in PD-MCI caregivers which was significantly lower than that of PDD caregivers but not significantly higher than PD-N carers. Levels of ZBI in the PD-MCI and PDD caregivers reported by Leroi (23.61 and 35.48) respectively, were similar to the differences found in the current study (22.00 and 29.33). One disparity compared to the current study was higher levels of burden reported by PD-N caregivers (20.00) in the Leroi study. Perhaps Leroi’s predominantly male caregivers found the caregiving role demanding in the early stages of PD, although previous research suggests female caregivers experience higher perceived burden [30] and we found no significant gender differences in the extent to which the current study’s PD-N male and female caregivers reported ZBI [*t*(49) = .88, *p* = .38]. Another possible explanation for the contrasting finding is that continuous involvement in the New Zealand Brain Research’s longitudinal study has provided a sense of support to the caregivers of PD-N patients involved in this research and has thereby lowered their feelings of burden. Also relevant is that the PD-MCI criteria used in the current study is associated with high levels of conversion to PDD over a four-year period [4].

The study by Szeto et al. [10] compared caregivers for PD-N and PD-MCI patients only. While the authors reported significantly lower quality of life scores with regard to physical health and interruptions to usual activities among PD-MCI caregivers, they mention that there was no difference in caregiver burden. They did not, however, report the actual levels of burden, but stated that the results were similar to those of Leroi et al. [9] These two studies used level I criteria to establish PD-MCI status, whereas the current study employed the more comprehensive level II criteria, which has been shown to be suitable in terms of stability and increased risk of progression to PDD [4]. Also, level I criteria may underestimate the proportion of PD-MCI patients and thus misclassify some PD-MCI patients as PD-N [31]. In another study of PD carers Lawson et al. [8] used a range of cognitive tests, though using modified Level II criteria, to identify PD-MCI patients. Lawson et al. reported that quality of life scores for PD-MCI carers were intermediate to that of caregivers for patients classified as PD-N (higher) and PDD (lower). Their results support the findings of the current study, which identified intermediate levels of distress among PD-MCI caregivers relative to caregivers of patients meeting PDD and PD-N classification. Unlike the Lawson study we were unable to find support that attentional deficits were related to caregiver distress.

Elevated burden among PDD caregivers in the current study compared with PD-N caregivers is consistent with previous research. Indeed 80% of PDD caregivers scored above the 21-point threshold on the ZBI, indicating mild to severe burden [13]. This threshold of burden was also frequent in PD-MCI caregivers (60%), and even apparent in a substantial number of caregivers of PD-N patients (30%) [11]. Given the high rates of burden irrespective of cognitive status, it is possible that neuropsychiatric and motor symptoms associated with PD and disease progression play a significant part towards negative caregiver outcomes. The significant effects of PD patient cognitive status nonetheless remained significant after controlling for neuropsychiatric symptoms and motor severity even though these features increased progressively with worsening cognition. Notably caregiver burden was also independent of disease duration. The difference in the number of hours spent caregiving between the three patient cognitive status groups suggests that even in the early stages of the disease the presence of impaired cognition places an additional load on caregivers. These observations lend support for the notion of PD-MCI as a clinical identity that has a significant impact not just on the person with PD but also the primary carer.

This is the first study to examine caregiver coping strategies as potential mediators of the relationship between patients’ cognitive status and caregiver burden in PD. The use of all three coping strategies increased as the patient’s cognitive status worsened. However, no evidence was found to suggest that the use of any of these coping strategies reduced caregiver burden in the current study. In fact, increased use of all three coping strategies was related to higher burden scores across the whole PD sample. For dysfunctional coping (disengaging from the situation or emotions) these findings are in line with other studies on dementia [14], and for problem-focused coping these findings add to a growing literature suggesting that responding with problem-focused strategies to situations that are beyond one’s control, such as when caring for an individual with a deteriorating illness, may not be effective [19]. For emotion-focused coping, however, these findings are not in line with studies of dementia caregivers showing that emotion-focused coping (managing one’s emotional response to stress) was associated with better caregiver outcomes (lower levels of anxiety and depression) [14]. Because of its correlational design the current study was unable to draw conclusions about causality. Although the three coping strategies mediated the relationship between cognitive status and caregiver burden, the reverse causation models (with burden mediating the relationship between cognitive status and coping) were also significant. The complex and possibly bidirectional relationship between caregiver outcomes and coping strategies was also illustrated in a longitudinal study of dementia caregivers by Cooper et al. [19]. They found that cross-sectionally caregiver burden was related to increased emotion-focused, problem-focused and dysfunctional coping (in line with findings from the current study), but that longitudinally an increased use of emotion-focused coping was related to lower anxiety.

### Limitations

Further evidence on burden and coping strategies in PD-MCI patients is needed because the current study had a relatively low number of participants and group sizes differed. Another limitation is the cross-sectional design. Longitudinal data could better assess the temporal course of caregiver outcomes and disentangle the directional causality between ZBI, cognitive impairment and coping strategies.

## Conclusions

The current study showed that caregivers of PD-MCI patients, known to have a high risk of dementia, often experience high levels of burden. PD without cognitive impairment is less often associated with caregiver burden, but this is also apparent even in a subset of these carers. The association between cognitive impairment and caregiver burden is relatively independent of neuropsychiatric and motor symptoms, disease duration and patient ability to engage in tasks of daily living. The findings add weight to the need for greater awareness of cognitive decline in PD patients and in particular to provide better support frameworks for caregivers before the onset of dementia. Increased support for caregivers may benefit patients and help delay the need for nursing home placement.

## Additional file

Additional file 1: Principal Component Factor Analysis of the Brief Coping Orientations for Problems Experienced (COPE). (PDF 2383 kb)
