# Supplementary material for: Caregiver burden is increased in Parkinson’s disease with mild cognitive impairment (PD-MCI)
Source: Transl Neurodegener. 2017 Jun 19;6:17. doi: 10.1186/s40035-017-0085-5 (PMC5474856; doi:10.1186/s40035-017-0085-5)
Supplement: Additional file 1: — Principal Component Factor Analysis of the Brief Coping Orientations for Problems Experienced (COPE). (PDF 2383 kb) [file 40035_2017_85_MOESM1_ESM.pdf]

Additional file

Factor Analysis of the Brief COPE

The factor analyses were completed using the Orthosim program [1]. Initially, principal components analysis with varimax rotation was undertaken to identify possible clusters among the individal items of the Brief COPE. The number of factors with eigenvalues greater than 1[2] and the results of the scree test [3] suggested that a 3 factor solution was optimal, consistent with the 3 factor solution identified by Carver and colleagues [4].

A previous study of dementia caregivers, which described problem-focused, emotion-focused and dysfunctional dimensions of the brief COPE [5], provided a target matrix for the current data. To examine whether these data could be rotated into maximal congruity with the dimensions used by Cooper and colleagues, an orthogonal procrustes procedure [6] was implemented. The procedure reports the 'target-comparison' fit as a series of congruence coefficients between each respective component factor from both matrices (Table A). The procedure also reports a 'mean solution congruence' which is the average congruence computed across all Brief COPE items, where each target item vector (factor) is compared to its counterpart in the comparison matrix.

Items loading on to factor 1 included the self-distraction, substance use, behavioural disengagement and venting subscale items from the dysfunctional dimension. Items loading onto factor 2 included the religion, acceptance and humour subscales and one items from the positive reframing subscale which formed the emotion-focused dimension. The emotional support subscale loaded more strongly to items from factor 3, the problem-focused subscale. It was felt that seeking emotional support could sit well in a dimension associated with finding solutions, therefore it remained in that dimension along with active coping, seeking instrumental support and planning subscale items. The overall solution congruence of .79 fell only just below the acceptable criterion of .8 [7]. The congruence coefficients between the corresponding target and input matrix component factors are provided in the first row of Table B and are slightly below the preferred criterion but were also considered acceptable for research purposes [7].

Internal Consistency

The Cronbach coefficient α was calculated for each of Carver's original subscale item pairings (Table B). All items pairs except positive reframing (.21) and denial (.49) exceeded Carver's criterion

of .5, although self-blame (.57) and behavioural disengagement (.56) failed to reach the conventionally accepted criterion of .7 [7].

Table A. Congruence coefficients and component-factor loadings from the maximally congruent orthogonal procrustes solution derived from a Principal Components factor analysis of the Brief COPE in a sample of PD patient caregivers (n=96).

	Factor 1 (Dysfunctional)	Factor 2 (Emotion-focused)	Factor 3 (Problem-focused)
Congruence coefficient	.76*	.67*	.84*
Self-Distraction 1	.62	-.06	.41
Self- Distraction 2	.51	-.06	.35
Denial 1	.25	.30	-.01
Denial 2	.32	.26	-.22
Substance Use 1	.64	-.35	.12
Substance Use 2	.60	-.17	.01
Behavioural Disengagement 1	.61	.06	-.08
Behavioural Disengagement 2	.55	.16	-.06
Venting 1	.56	.17	.22
Venting 2	.60	.44	.06
Self-Blame 1	.37	.16	.20
Self-Blame 2	.09	.25	-.04
Emotional Support 1	.18	.18	.69
Emotional Support 2	.12	.25	.64
Positive Reframing 1	-.02	.20	.31
Positive Reframing 2	.22	.64	.07
Humour 1	.05	.66	-.06
Humour 2	.14	.55	-.11
Acceptance 1	.03	.41	.22
Acceptance 2	.03	.59	.24
Religion 1	.21	.50	-.04
Religion 2	.21	.51	-.04
Active Coping1	.30	.10	.57
Active Coping2	.33	.22	.66
Instrumental Support1	-.06	.23	.74
Instrumental Support2	-.06	.54	.56
Planning 1	.23	.33	.52
Planning 2	.19	.52	.49

Table B. Internal consistency (Cronbach α) of Brief COPE subscale item pairs in a PD caregiver sample (n=96)

**1. Active Coping**	**α = .76**	• I've been concentrating my efforts on doing something about the situation I'm in.<br>• I've been taking action to try to make the situation better.
**2. Emotional Support**	α = .81	• I've been getting emotional support from others.<br>• I've been getting comfort and understanding from someone.
**3. Instrumental Support**	α = .76	• I've been getting help and advice from other people.<br>• I've been trying to get advice or help from other people about what to do.
**4. Planning**	α = .70	• I've been trying to come up with a strategy about what to do.<br>• I've been thinking hard about what steps to take.
**5. Humour**	α= .79	• I've been making jokes about it.<br>• I've been making fun of the situation.
**6. Venting**	α = .72	• I've been saying things to let my unpleasant feelings escape.<br>• I've been expressing my negative feelings.
**7. Acceptance**	α = .70	• I've been accepting the reality of the fact that it has happened.<br>• I've been learning to live with it.
**8. Positive Reframing**	α= .21	• I've been trying to see it in a different light, to make it seem more positive.<br>• I've been looking for something good in what is happening.
**9. Religion**	α= .89	• I've been trying to find comfort in my religion or spiritual beliefs.<br>• I've been praying or meditating.
**10. Substance Use**	α= .76	• I've been using alcohol or other drugs to help me get through it.<br>• I've been using alcohol or other drugs to help me get through it.
**11. Behavioural Disengagement**	α= .56	• I've been giving up trying to deal with it.<br>• I've been giving up the attempt to cope
**12. Denial**	α = .49	• I've been saying to myself "this isn't real".<br>• I've been refusing to believe that it has happened.
**13. Self-Blame**	α = .57	• I've been criticizing myself.<br>• I've been blaming myself for things that happened.
**14. Self-Distraction**	α = .84	• I've been turning to work or other activities to take my mind off things.<br>• I've been doing something to think about it less, such as going to movies, watching TV, reading, daydreaming, sleeping or shopping.

Bibliography

1. Barrett, P. T. (2006). Orthosim 2 v .01. Auckland: Paul Barrett.
2. Kaiser H. F. (1960) The application of electronic computers to factor analysis. *Educational and Psychological Measurement*, *20*, 141-151.
3. Cattell RB. (1966). The scree test for the number of factors. *Multivariate Behavioural Research*, *1,* 245-276.
4. Carver, C.S., Scheier, M.F. and Weintraub, J.K. (1989). Assessing coping strategies: a theoretically based approach. *J Pers Soc Psychol*, *56(2),* 267-83.
5. Cooper, C., et al., *Coping strategies, anxiety and depression in caregivers of people with Alzheimer's disease.* Int J Geriatr Psychiatry, 2008. **23**(9): p. 929-36.
6. Schonemann P. H. (1968). On two-sided orthogonal Procrustes problems. *Psychometrika* *33*(1):19-33.
7. Streiner D. L. (2003). Starting at the beginning: an introduction to coefficient alpha and internal consistency. *J Pers Assess 80*(1):99-103.
